# Long-Term Risk of Acute Myocardial Infarction in Patients With a Transient Ischemic Attack: A Danish Nationwide Cohort Study

**DOI:** 10.1161/STROKEAHA.123.045605

**Published:** 2024-12-27

**Authors:** Habibullah Safi, Søren Lund Kristensen, Rikke Sørensen, Christina Kruuse, Søren Paaske Johnsen, Gunnar Gislason, Christian Torp-Pedersen, Lars Køber, Emil L. Fosbøl, Naja Emborg Vinding

**Affiliations:** 1 Department of Cardiology, Copenhagen University Hospital, Rigshospitalet, Denmark (H.S., S.L.K., R.S., L.K., E.L.F., N.E.V.).; 2 Department of Neurology, Copenhagen University Hospital, Herlev and Gentofte, Denmark (C.K.).; 3 Department of Clinical Medicine, Danish Center for Clinical Health Services Research, Aalborg University, Denmark (S.P.J.).; 4 Danish Heart Foundation, Research Department, Copenhagen, Denmark (G.G.).; 5 Department of Cardiology, Nordsjællands Hospital, Hillerød, Denmark (C.T.-P.).; 6 Department of Public Health, University of Copenhagen, Denmark (C.T.-P.).

**Keywords:** Denmark, ischemic attack, transient, myocardial infarction, registries, stroke, ischemic

## Abstract

**BACKGROUND:**

Sparse information regarding the long-term risk of acute myocardial infarction (MI) following a transient ischemic attack (TIA) emphasizes further research to guide preventive strategies and risk stratification in patients with a TIA.

**METHODS:**

We conducted a nationwide cohort study to investigate the 5-year risk of MI and all-cause mortality in patients with a first-time TIA. Patients with a first-time TIA were identified in the Danish Stroke Registry (2013–2020), matched on age, sex, and calendar year (1:4) with the general population and (1:1) with patients with first-time ischemic stroke. The 5-year risks of MI and all-cause mortality were estimated by the Aalen-Johansen and Kaplan-Meier estimators. The groups were compared using Cox regression, while adjusting for cardiovascular comorbidities.

**RESULTS:**

We identified 21 743 patients with TIA, 86 972 matched individuals from the general population, and 21 743 matched control patients with ischemic stroke. Median age was 70 (25th to 75th percentile, 60–78) years; 52% were male. Comorbidity burden was the lowest in general population controls, intermediate in patients with TIA, and the highest in patients with ischemic stroke. The 5-year risk of MI was 2.0% in patients with TIA, 1.5% in the general population (*P*<0.001), and 2.2% in the ischemic stroke population (*P*<0.001). After adjustment, these differences in MI rate were similar (TIA versus general population; hazard ratio, 0.99 [95% CI, 0.98–1.02] and TIA versus ischemic stroke; hazard ratio, 0.99 [95% CI, 0.96–1.01]). The 5-year risk of mortality was 17.0% in patients with TIA compared with 14.0% in the general population (*P*<0.001) and 27.0% in ischemic stroke population (*P*<0.001). The differences in mortality persisted following adjustments for patients with TIA versus general population (hazard ratio, 1.25 [95% CI, 1.19–1.31]) and for patients with TIA versus ischemic stroke (hazard ratio, 0.43 [95% CI, 0.41–0.46]).

**CONCLUSIONS:**

Patients with first-time TIA had a low 5-year incidence of MI, which was not significantly different from that of the general population and patients with first-time ischemic stroke after adjustments for comorbidities. However, patients with TIA had a 25% higher all-cause mortality rate than the general population, which was not readily explained by MI risk. Hence, the findings do not endorse the need to raise further awareness regarding MI in patients with TIA.

Recurrent cerebral and cardiovascular events in patients with a transient ischemic attack (TIA) and ischemic stroke have far-reaching consequences, frequently cited as the leading cause of chronic disability and death.^1–9^ Despite the recent attention toward the cardiovascular complication of acute myocardial infarction (MI), the long-term risk of MI following TIA still remains unknown. This uncertainty presents an opportunity to enhance public health by further investigating and addressing this potential risk.

Prior reports on the annual risk of MI following TIA and stroke exhibited significant variations, with estimates ranging from 1.0% to 6.5%. The long-term risk (5–10 years of follow-up) of all-cause mortality ranged from 10% to 42% in the last 2 decades.^1–8^ In addition, current population-based information on the risk of MI and mortality in patients with TIA is sparse. Most crucially, comparing patients with TIA and the general population will provide insight into whether patients with TIA should be considered at high risk of MI.^2,4^ Recent advancements and continuous progress in secondary prevention of cerebral and cardiovascular events, along the long-term management of critical modifiable risk factors, have contributed to reducing cardiovascular mortality and risk in patients with TIA.^1,3,4,7,8,10–13^ However, there may still be potential for reducing residual cardiovascular risk in patients with TIA and stroke, which multiple studies have emphasized.^1,3–5,10^

To address these knowledge gaps, we examine the long-term risk of MI and all-cause mortality in patients with a first-time TIA and compare it to both the general population and matched control patients with first-time ischemic stroke.

## Methods

This study adheres to the STROBE guidelines (Strengthening the Reporting of Observational Studies in Epidemiology; Supplemental Material).

### Data Availability

Given the sensitive nature of the personal data involved in this research, raw data are not available in accordance with data protection regulations at Statistics Denmark. For more information about the analysis, please contact the corresponding author.

### Data Sources

Danish citizens are provided with a unique and permanent civil identifier that allows linkage to multiple nationwide registries. In this study, the following national registries were linked:

The Danish Stroke Registry holds information on all patients with acute stroke since 2003 and TIA since 2013 to monitor and improve stroke and TIA care.^14^

The Danish National Patient Registry holds all information about hospital admissions since 1977 and outpatient contacts since 1995. The data are coded according to the *International Classification of Diseases*, *Eighth and Tenth Revisions*, surgical procedures.^15^

The Danish National Prescription Registry has held information on all claimed drug prescriptions since 1995.^15^

The Danish Civil Registration System contains sex, birth date, emigration, and vital status information.

The Danish Registry of Causes of Death contains information on the date, place, and cause of death.^16^

Table S1 provides detailed *International Classification of Diseases*, *Eighth and Tenth Revisions*, codes for all comorbidities and Table S2 ATC codes for the pharmacotherapy included in the study (Tables S1 and S2).

### Study Population and Inclusion Criteria

In the Danish Stroke Registry, we identified all patients with first-time TIA and patients with first-time ischemic or unspecified stroke between January 1, 2013, and December 31, 2020. The admission dates for TIA or stroke were defined as the index date. Individuals from the general population were found in the Danish Civil Registration System and given randomly generated index dates based on a pool of the TIA index dates.

**Figure 1.**
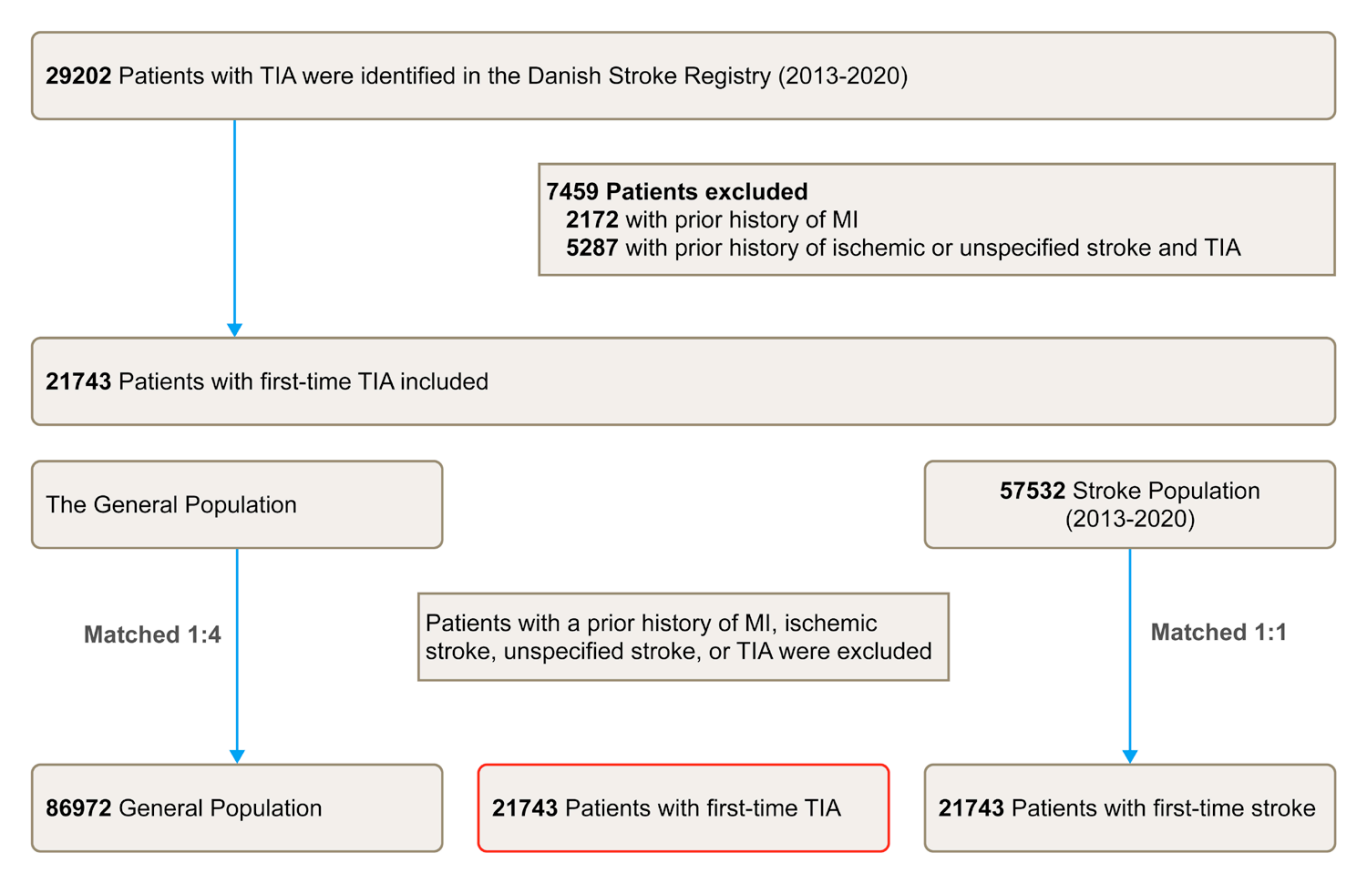
**Flowchart of selection of the study population.** AMI indicates acute myocardial infarction; and TIA, transient ischemic attack.

Patients with TIA and the controls were included if they met the following inclusion criteria: (1) above 18 years of age and (2) had no history of MI, ischemic or unspecified stroke, or TIA. By use of exposure density matching, patients with TIA were matched on age, sex, and index calendar year, with the general population in a ratio of 1:4 and the stroke population in a ratio of 1:1 (Figure 1).

The patient’s medical history was obtained using the Danish National Patient Registry before the index date. Pharmacotherapy was assessed by the Danish National Prescription Registry 6 months before the index. We identified patients with diabetes and hypertension based on claimed drug prescriptions.^17^ It is important to note that diagnosing these conditions through claimed prescription may likely lead to an underestimation of the prevalence of these comorbidities. Prior bleeding in Table 1, baseline characteristics of the study population, refers to any bleeding-related condition 6 months before the index date (Table 1). This includes, but is not limited to, intracerebral hemorrhage, gastrointestinal ulcers with bleeding, and other similar conditions (Tables S1 and S2). Lifestyle factors for patients with TIA and control ischemic stroke population were obtained from the Danish Stroke Registry.

**Table 1.**
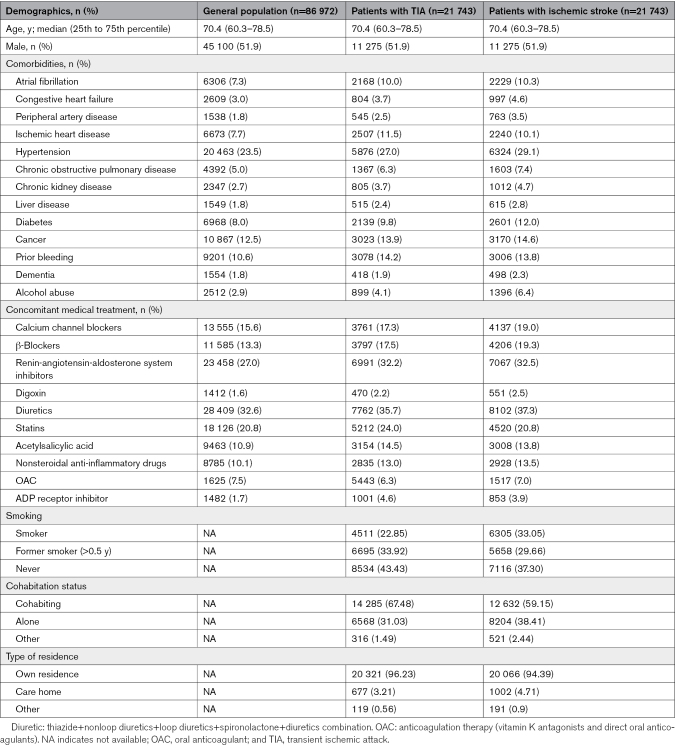
Baseline Characteristics of the Study Population

### Validation of TIA Diagnosis

Vinding et al^18^ validated the diagnosis of TIA with positive predictive value (PPV) of 78.8%. Registered TIA events (n=200) were validated by a stroke neurology professor and a neurology trainee, each evaluating a random sample of 100 patients independently and blind from the Danish Stroke Registry. If there was uncertainty in the diagnosis, a third stroke specialist provided the final assessment.^18^

### Secondary Prophylaxis Medication

The Danish National, European, and American guidelines advocate for a comprehensive long-term secondary prophylaxis medication approach in patients who had a TIA or stroke. This strategy includes antithrombotic therapy and the management of modifiable risk factors such as lipid-lowering, antihypertensive, and antidiabetic agents. Its objective is to prevent recurrent vascular events.^11,12,19^ The long-term guideline for antiplatelet strategy recommends acetylsalicylic acid alone, adenosine phosphate inhibitor, and phosphodiesterase inhibitor alone or in combination with acetylsalicylic acid. In Denmark, the protocol for dual antiplatelet therapy adheres to guidelines derived from the POINT and CHANCE trials, advocating for a 21-day treatment regimen.^19^ Oral anticoagulation therapy (vitamin K antagonists and direct oral anticoagulants) is indicated in patients with TIA and atrial fibrillation. Individuals who died or emigrated within 90 days after discharge were excluded. In the secondary prevention prophylaxis analysis, we included only prescriptions issued after the event with a 90-day grace period for assessment. Details of the specific medications included can be found in Table 2 and Table S2. We defined an increase in antihypertensive therapy solely as the addition of a new antihypertensive medication.

**Table 2.**
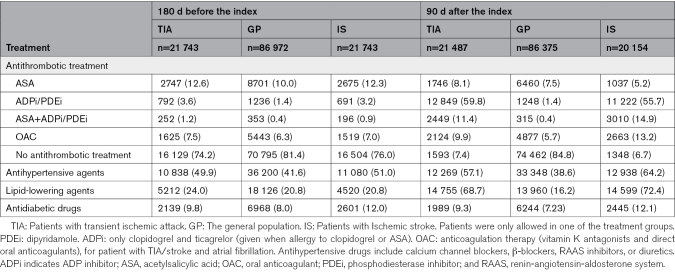
Antithrombotic and Risk Control Treatment

### Follow-Up and Outcomes

The primary outcome was hospital admission with MI (Table S1). The secondary outcome was all-cause mortality. Patients were followed from the index date until whichever came first: occurrence of the outcomes, maximum of 5 years of follow-up, end of study (December 31, 2020), and emigration.

### Statistical Analysis

Categorical variables were reported as frequency and percentages. Continuous variables were reported as median with 25th to 75th percentiles. χ^2^ tests were used for testing differences in categorical variables. Kruskal-Wallis tests were used for testing differences between continuous variables. The crude cumulative incidence of MI per group was estimated using the Aalen-Johansen estimator, while all-cause mortality was assessed as a competing risk. Gray test was used to assess the difference between the groups. The crude cumulative incidence of all-cause mortality was estimated using the Kaplan-Meier estimator. Log-rank test was used to assess the difference between the curves. Conditional on matching, multivariable Cox regression analysis was used for adjusting for cardiovascular comorbidities: atrial fibrillation, diabetes, peripheral artery disease, heart failure, hypertension, chronic kidney disease, alcohol abuse, bleeding, and prior use of statins. Cox proportional hazard assumption and interaction with sex and age were tested, and assumptions underlying the models were considered valid.

The statistical analyses were performed using SAS, version 9.4, and R. *P*<0.05 was considered statistically significant.

### Ethical Approval

The Danish Data Agency approved this project with the numbers P-2019-191. Every individual’s unique and permanent civil identifier is encrypted and cannot be identified, and registry-based studies do not need ethics approval.

## Results

### Study Population and Baseline Characteristics

We identified 21 743 patients with first-time TIA, 86 972 control individuals from the general population, and 21 743 control patients with first-time ischemic stroke (Figure 1). The baseline characteristics are summarized in Table 1. The median age was 70 (25th to 75th percentile, 60–78) years, and 52% were male. The comorbidity burden was the lowest in the general population, intermediate in patients with TIA, and the highest in the ischemic stroke population. The 3 most common comorbidities in all the populations were hypertension, cancer, and prior bleeding. The 3 standard concomitant medical treatments in all the populations were diuretics, renin-angiotensin-aldosterone system inhibitors, and lipid-lowering agents. Patients with TIA had a higher proportion with prior use of statins (24.0% versus 20.8% versus 20.8%), acetylsalicylic acid (14.5% versus 10.9% versus 13.8%), adenosine phosphate inhibitor, and phosphodiesterase inhibitor (4.6% versus 1.7% versus 3.9%), compared with the general population and patients with ischemic stroke 180 days before baseline.

### Risk of MI

**Figure 2.**
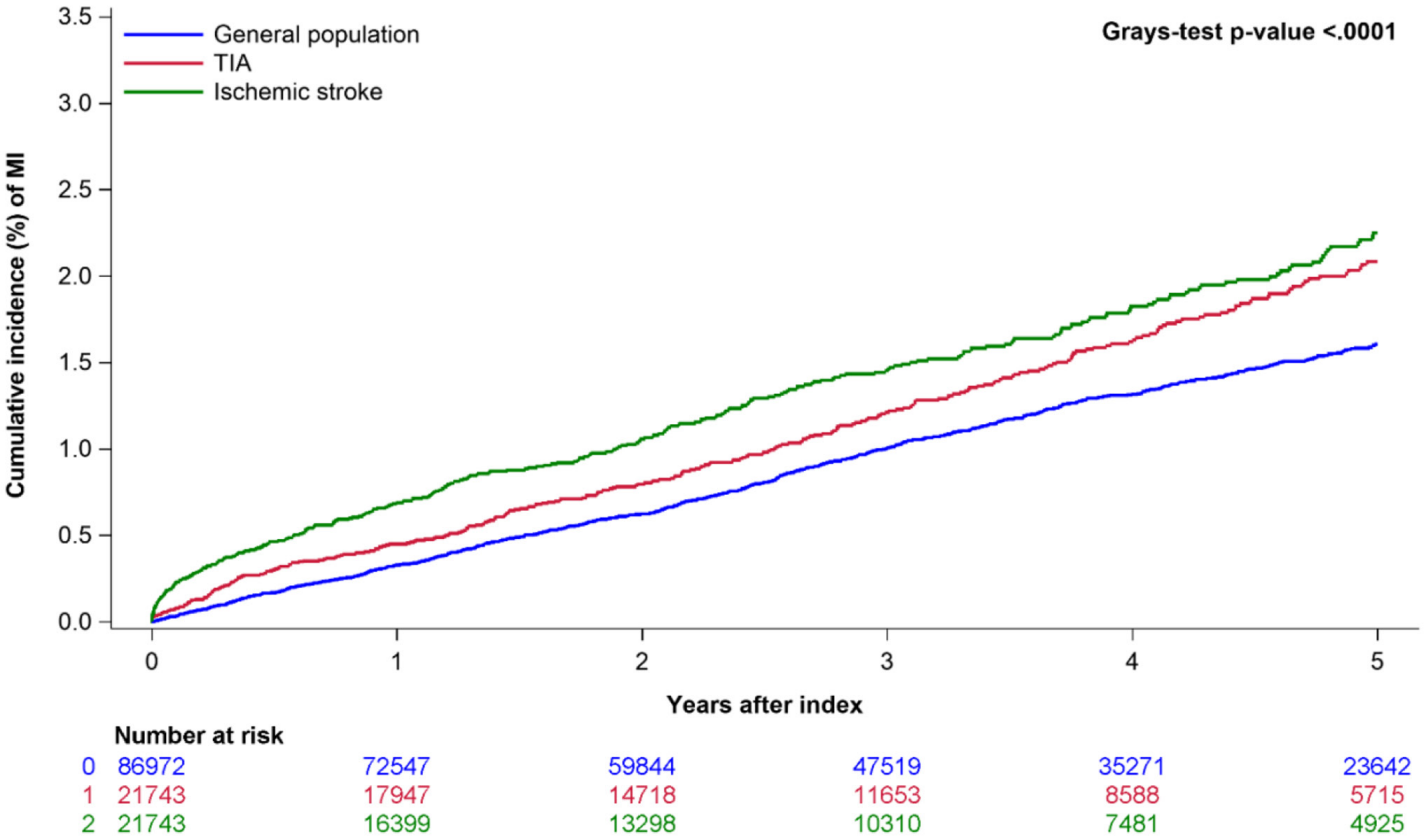
Five-year cumulative incidence (%) of acute myocardial infarction (MI) in patients with a transient ischemic attack (TIA; red), ischemic stroke population (green), and the general population (blue).

**Figure 3.**
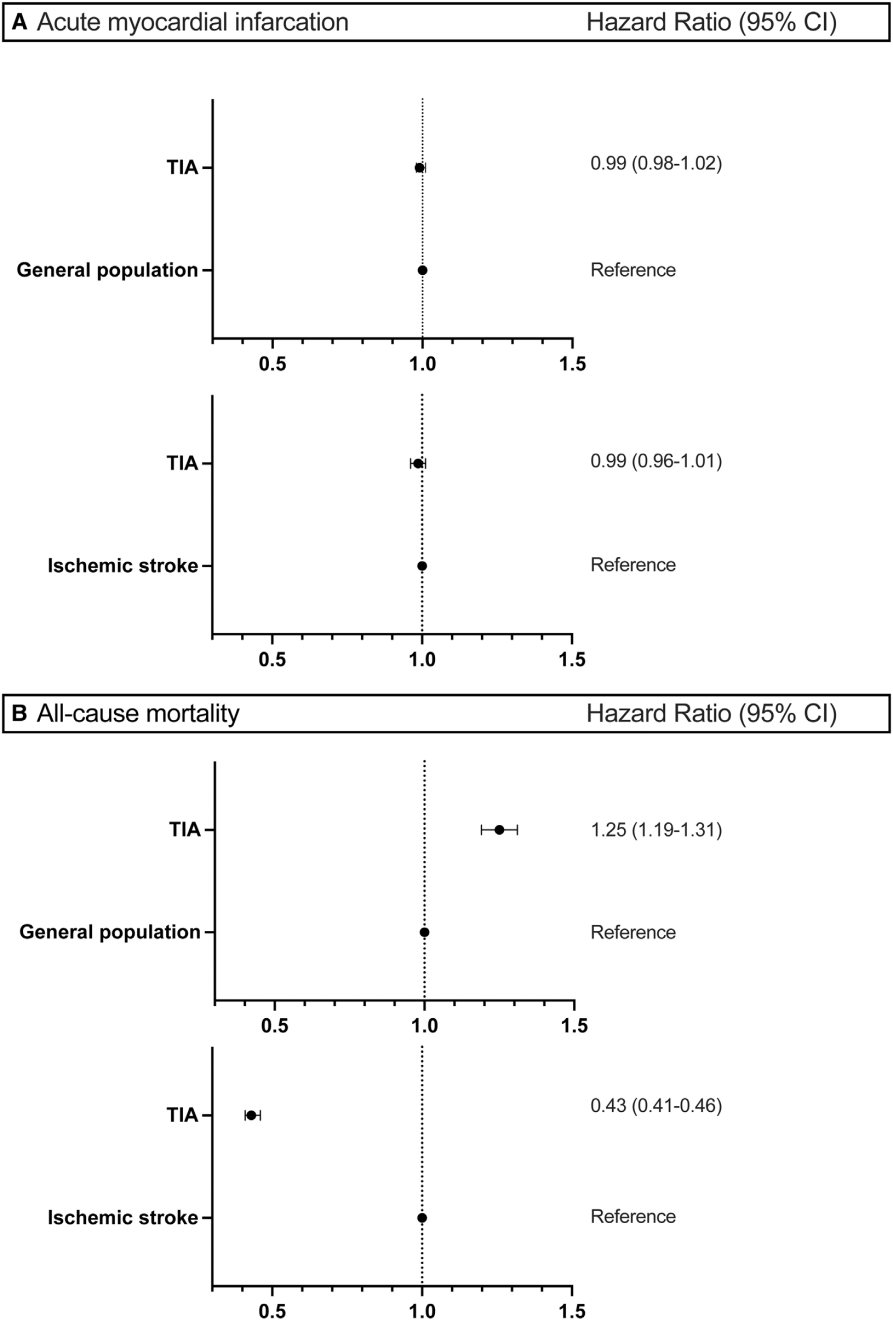
**Forest plot with adjusted hazard ratio (HR).** Forest plot with adjusted HR for (**A**) acute myocardial infarction and (**B**) all-cause mortality. This model has been adjusted for atrial fibrillation, diabetes, peripheral artery disease, heart failure, chronic kidney disease, harmful alcohol use, prior bleeding, and prior use of statins. Conditional on matching. TIA indicates transient ischemic attack.

The 5-year cumulative incidence of MI was 2.0%, 1.5%, and 2.2% for patients with TIA, the general population, and the ischemic stroke population, respectively (*P*<0.0001; Figure 2). After adjustment, we found no difference in incidence for patients with TIA compared with the general population (unadjusted hazard ratio [HR], 0.99 [95% CI, 0.98–1.01]; adjusted HR, 0.99 [95% CI, 0.98–1.01]) or the ischemic stroke population (unadjusted HR, 0.98 [95% CI, 0.95–1.01]; adjusted HR, 0.99 [95% CI, 0.96–1.01]; Figure 3).

### All-Cause Mortality

**Figure 4.**
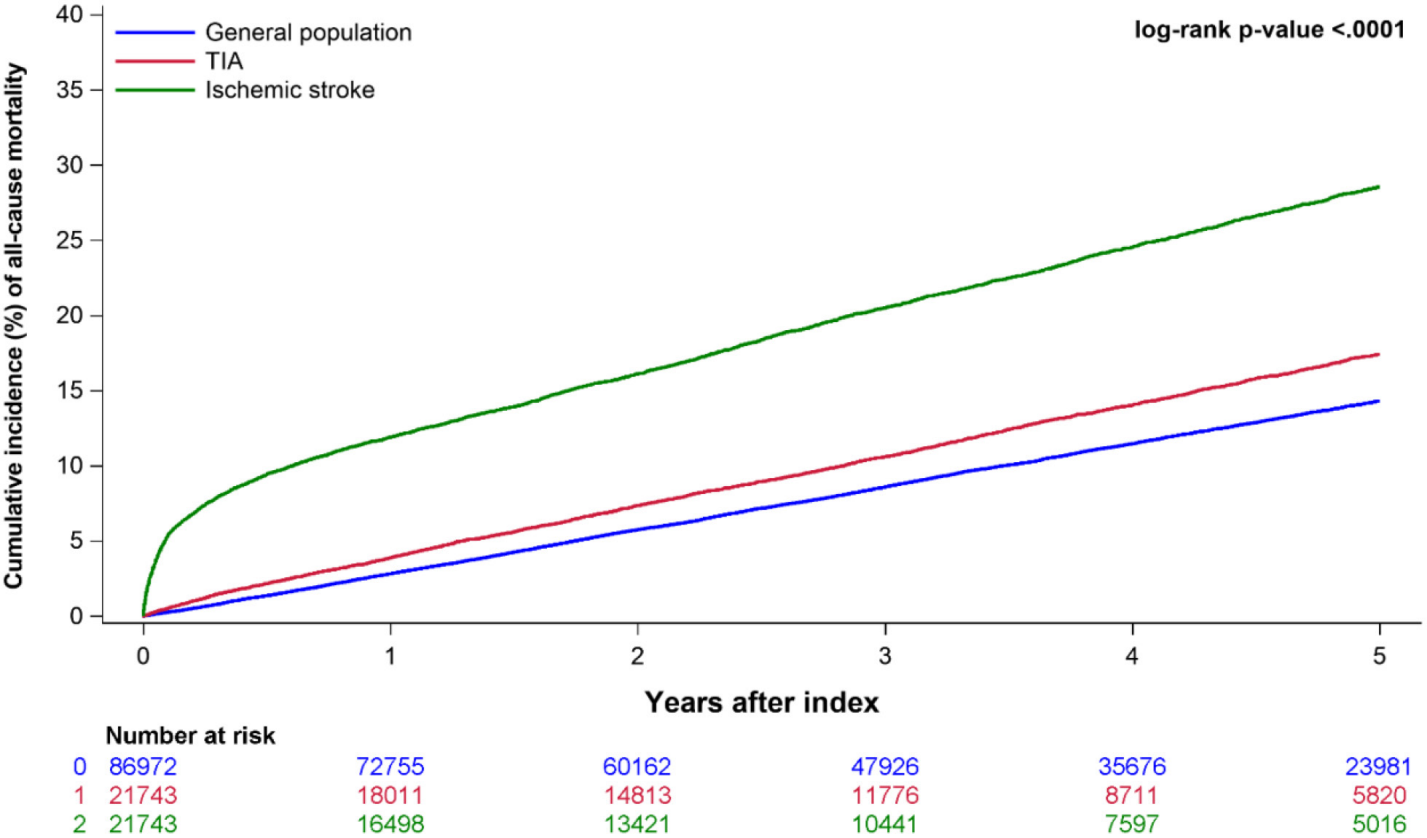
Five-year cumulative incidence (%) of all-cause mortality in patients with a transient ischemic attack (TIA; red), ischemic stroke population (green), and the general population (blue).

The 5-year cumulative incidence of all-cause mortality was higher in patients with TIA compared with the general population (17.0% versus 14.0%; *P*<0.0001; unadjusted HR, 1.29 [95% CI, 1.23–1.35]; adjusted HR, 1.25 [95% CI, 1.19–1.31]) but lower than the ischemic stroke population (27.0%; *P*<0.0001; unadjusted HR, 0.42 [95% CI, 0.40–0.45]; adjusted HR, 0.43 [95% CI, 0.41–0.46]; Figures 3 and 4).

### Secondary Prophylaxis Medication

The antithrombotic and risk control treatment is summarized in Table 2. One hundred eighty days before the index date, the majority in each of the 3 populations did not receive antithrombotic treatment, antihypertensive or lipid-lowering agents. Three months post-discharge, 92.6% of patients with TIA and 93.3% of patients with ischemic stroke received antithrombotic treatment and increased antihypertensive agents (57.1% and 64.2%) and lipid-lowering agents (68.7% and 72.4%). Majority of patients in both groups received adenosine phosphate inhibitor and phosphodiesterase inhibitor followed by acetylsalicylic acid.

Overall, 3 months post-discharge, patients with TIA had an absolute 66.8% increase in antithrombotic treatment, 7.2% in antihypertensive medications, and 44.7% in lipid-lowering agents compared with baseline, while patients with ischemic stroke had 69.3%, 13.2%, and 51.6%, respectively.

## Discussion

This study yielded 3 primary findings. First, the 5-year cumulative incidence of MI in a patient with first-time TIA was around 2%, which after adjustment for other risk factors was similar to the rate of MI in patients with first-time ischemic stroke and the general population. Second, 3 months post-discharge, patients with TIA had an absolute 66.8% increase in antithrombotic treatment, 7.2% in antihypertensive medications, and 44.7% in lipid-lowering agents compared with baseline, while patients with ischemic stroke had 69.3%, 13.2%, and 51.6%, respectively. Third, the 5-year cumulative incidence of all-cause mortality in patients with first-time TIA was 17% compared with 14% in the general population of all-cause mortality and 27% in the first-time ischemic stroke population, which held true in the adjusted models.

The 5-year cumulative incidence of MI at 2.0% in patients with TIA in our study is comparatively lower than previously reported in prior studies, with had an annual incidence of MI up to 6.5%.^1–6,10^ However, in a recent systematic review and meta-analysis of 58 studies, the annual risk of MI had significantly decreased over time (1970–2010) and the annual risk of MI was higher in studies restricted to patients with a history of coronary artery disease than in patients with no history of coronary artery disease.^2^ Likewise, the same trend of lower risk of cardiovascular outcome after TIA or minor stroke was found by researchers in the TIAregistry.org project (n=3847) than previously reported in historical cohorts.^1,10^ The following differences might have contributed to the low risk of MI found in this study compared with most prior studies,^1,2,10^ including updated nationwide data from 2013 to 2021; a separated TIA and stroke population; patients and control individuals with no prior coronary artery disease, TIA, or stroke; a primary outcome consisting of only MI and most importantly a higher significant proportion of patients treated secondary prophylaxis medication. Our study yielded that 3 months post-discharge, 92.6% of patients with TIA and 93.3% of patients with ischemic stroke received antithrombotic treatment and an increased prescription of antihypertensive medications (57.1% and 64.2%) and lipid-lowering agents (68.7% and 72.4%). Most patients in both groups received ADP inhibitor monotherapy followed by acetylsalicylic acid. The similarity is seen in the TIAregistry.org project, where at 1 year post-discharge, 78.0% of patients with TIA or minor stroke received antithrombotic treatment, 71.1% received antihypertensive medications, and 67.8% received statins compared with the baseline of 27.7%, 55.1%, and 27.8%, respectively.^1^ Therefore, it is plausible that the observed low risk and rate of MI in this study may reflect recent advancements in secondary prevention of cerebral and cardiovascular events, alongside sustained progress in managing key modifiable risk factors, both of which likely contribute to reduced cardiovascular mortality and risk in patients with TIA.^1,3,4,7,8,10–13^

While in our study, the risk of MI did not differ between patients with first-time TIA, first-time ischemic stroke, and the general population in adjusted models, 2 studies found the opposite. Burns et al^4^ found that the HR for patients with TIA (n=388) compared with the general population was 2.1 (95% CI, 1.5–2.8). Researchers in the Ontario Stroke Registry project compared patients (n=26 366) without early adverse complications after TIA and stroke to the general Ontario population in a ratio of 1:10.^3^ In the study, patients with TIA had a significantly higher 5-year risk of MI compared with the general population (HR, 1.6 [95% CI, 1.4–1.8]). Furthermore, the patients had a significantly higher HR for cardiovascular and cerebrovascular events during early and late follow-up periods.^3^ Differences in study setups may explain the differences in results on MI risk among patients with TIA. First, in contrast to our study, the 2 studies mentioned above included patients with TIA with prior MI, TIA, or stroke history. Second, the study by Burns et al was based on combined data from the Rochester Epidemiology Project for TIA from 1985 to 1994 and MI from 1979 to 2006. The Ontario study was based on data from 2003 to 2013.^3,4^ Consistent improvement in the secondary prevention and long-term management of risk factor controls throughout the years may lower the risk of MI.^1,3,4,7,8,10–13^ On a side note, the incidence of MI in the Ontario study might be higher if they had included the 11 681 (30.5%) patients with TIA or stroke who experienced an adverse complication within 90 days of discharge and, similarly, if our study had included patients with a history of TIA, stroke, and MI.^2,3^

Prior studies on this topic have presented 5- and 10-year long-term mortality risks of up to 42%.^1,3,5,10^ The Ontario study reported a 5-year cumulative incidence of mortality of patients with TIA and stroke and the matched controls of 22.9%, 27.9%, and 16.9%, respectively. In the Ontario study, patients with TIA and stroke had a relative 5-year mortality (HRs, 1.4 [95% CI, 1.3–1.5] and 1.8 [95% CI, 1.7–1.8], respectively), compared with the general population as reference.^3^ Differences in mortality rate may be explained by the variations between the studies, as mentioned earlier in the discussion regarding the risk of MI. Although it would have been interesting to investigate the cause of death, whether it was of vascular or nonvascular nature, we are limited by the lack of validation and low PPV of this information in the Danish Registry of Cause of Death.^16^ In the LiLAC cohort study (Life Long After Cerebral Ischaemia; n=2447), 1336 patients with TIA or stroke died with vascular death (62%) as the leading cause of death.^5^ In another study (n=10 981), the most frequent causes of death were cerebrovascular diseases (24.1%), pneumonia (22.6%), heart disease (18.1%), and cancer (11.0%).^20^ In the TIAregistry.org project study, 96 of 376 deaths were attributed to cardiovascular causes (44 fatal strokes and 3 fatal AMI) in a 5-year follow-up period.^1^ Recurrent strokes and stroke-related events were among the most frequent causes of rehospitalization in patients with TIA and stroke.^21,22^

In summary, this study does not emphasize the necessity for specific focus on MI in patients with TIA, as it may not be the primary cause for the observed higher mortality rates compared with the general population. Last, despite the advancement made in the secondary prevention of recurrent cerebral and cardiovascular events, along the long-term management of critical modifiable risk factors, multiple studies still have highlighted the potential for further reducing cardiovascular events and mortality through improvements in these areas.^1,3–5,7,8,10–12^ Future studies or interventions examining short- and long-term secondary nonpharmacological/pharmacological prevention and supportive services in patients with TIA and stroke would be welcomed. Progress in newer studies and trials in secondary prophylaxis strategy with anti-inflammatory medication following TIA, stroke, and MI is also worth noticing and following in the future as it might provide exciting data.^23–26^ To enhance management, to enhance the follow-up of patients with TIA, and to reduce the risk of subsequent cerebrovascular events, we underscore the importance of adhering to guidelines. These guidelines advocate for a comprehensive and multidisciplinary approach that includes lifestyle changes, pharmacological interventions, and possibly surgical options tailored to individual patient needs.^1,3–5,7,8,10–12^

### Strengths and Limitations

The main strength of this study is the completeness of data in the multiple nationwide Danish registries with the possibility of long-term follow-up. All Danish residents are provided by the health care system with equal access to health care, which means minimal demographic or socioeconomic bias. We found a PPV of 78.8%, which was higher compared with the diagnosis in the Danish National Patient Registry. Prior studies have validated for the diagnosis of MI with a PPV ≥90% and stroke with a PPV of 90%.^14,15,27^

The main limitation of this study is the observational study design. Therefore, only associative conclusions can be drawn, and causal interpretations should be made with caution. The diagnosis of TIA can be complicated due to the transient nature of the symptoms, often resolving by the time patients arrive at the hospital. Additionally, the presence of numerous TIA mimics and chameleons adds further complexity to the diagnostic process. Vinding et al validated the diagnosis of TIA to 78.8%.^18^ Therefore, it is crucial to acknowledge that with a PPV of 78.8%, there will be some patients in the study population who may had received a false-positive TIA diagnosis. Moreover, the exclusion of the 7 inconclusive cases post-adjudication in the validation process may have influenced the PPV, potentially affecting the precision of the study’s finding and main conclusions on the risk of MI. Also, as most of the patients were only validated by 1 rater, it was not possible to calculate interrater variability. Still, TIA care has in Denmark to a high extent been centralized to specialized TIA clinics staffed with neurologists supporting the likelihood of high diagnostic accuracy. Another limitation of this study is that the primary outcome of MI did not include out-of-hospital MI deaths, due to limitations in the Danish Registry of Cause of Death, as mentioned in the discussion. The Danish Stroke Registry has no information about the etiological classifications of stroke, which would have made it possible to divide in subtype classification, for example, using the TOAST (Trial of ORG 10172 in Acute Stroke Treatment) criteria, to investigate the risk difference in MI among the subtypes. Regarding secondary antithrombotic prophylaxis, we only have information on whether patients redeemed a prescription within the first 3 months; however, we lack data on their compliance.

### Conclusions

Patients with first-time TIA had a low 5-year incidence of MI, which was not significantly different from that of the general population and patients with first-time ischemic stroke after adjustments for comorbidities. However, patients with TIA had a 25% higher all-cause mortality rate than the general population, which was not readily explained by MI risk. Hence, the findings do not endorse the need to raise further awareness regarding MI in patients with TIA.

## Article Information

### Sources of Funding

This study was supported by a scholarship grant for students.

### Disclosures

Dr Kristensen reports advisory board honoraria from Bayer and AstraZeneca. Dr Johnsen reports consultant work for Bristol Myers Squibb and Pfizer and institutional grants from Bristol Myers Squibb, Pfizer, and Novo Nordisk. Dr Kruuse reports compensation from the Novo Nordisk Foundation and from Bayer Healthcare. Dr Torp-Pedersen reports grants for studies from Bayer and Novo Nordisk. Dr Køber reports speaker honoraria from Novo Nordisk, Novartis, AstraZeneca, Boehring, and Bayer. Dr Fosbøl reports grants from Novo Nordisk Fonden. Dr Sørensen reports compensation from Novo Nordisk for data and safety monitoring services. None of the disclosures mentioned are related to this study. The other authors report no conflicts.

### Supplemental Material

Tables S1–S2

STROBE Checklist
